# The Aachen ACLF ICU score predicts ICU mortality in critically ill patients with acute-on-chronic liver failure

**DOI:** 10.1038/s41598-024-82178-0

**Published:** 2024-12-16

**Authors:** Maike R. Pollmanns, Bastian Kister, Samira Abu Jhaisha, Jule K. Adams, Elena Kabak, Jonathan F. Brozat, Carolin V. Schneider, Philipp Hohlstein, Tony Bruns, Lars Küpfer, Christian Trautwein, Alexander Koch, Theresa H. Wirtz

**Affiliations:** 1 Medical Department III, RWTH Aachen University Hospital, Pauwelsstraße 30, 52074 Aachen, Germany; 2 Institute for Systems Medicine with Focus on Organ Interaction, RWTH Aachen University, Aachen, Germany; 3 Department of Hepatology and Gastroenterology, Charité-Universitätsmedizin Berlin, Campus Virchow-Klinikum (CVK) and Campus Charité Mitte (CCM), Berlin, Germany; 4 Leibniz Research Centre for Working Environment and Human Factors at the TU Dortmund (IfADo), Dortmund, Germany

**Keywords:** ACLF, Machine learning, Prognosis, Mortality, Liver cirrhosis, Prognosis

## Abstract

Acute-on-chronic liver failure (ACLF) defines a heterogeneous syndrome involving acute decompensation in patients with pre-existing liver disease accompanied by (multi-)organ failure. This study aimed to develop a simple, reliable machine learning (ML) model to predict mortality in ACLF patients receiving intensive care unit (ICU) treatment. Data from 206 patients admitted to the ICU at RWTH Aachen University Hospital between 2015 and 2021 were retrospectively analyzed with ICU mortality as the primary outcome. An ICU mortality prediction model was developed by logistic regression and validated by 5-fold cross validation. Performance metrics were assessed to evaluate the model’s accuracy and compare to existing mortality scores. ICU mortality was 60%. The chronic-liver-failure-consortium ACLF score (CLIF-C ACLFs) was the best predictor of ICU mortality. ML generated seven models using five to thirteen features. The best-performing model included CLIF-C ACLFs, number of organ failures, Horovitz quotient (FiO_2_/PaO_2_), FiO_2_ and lactate. The newly developed Aachen ACLF ICU (ACICU) score demonstrated exceptional predictive accuracy for ICU mortality (AUROC 0.96), underscoring its potential for mortality and futility assessment in critically ill ACLF patients complementing existing prognostic tools. The ACICU score www.acicu-score.com is an easy-to-use tool for predicting ICU mortality in patients with ACLF offering high predictive performance.

**Supplementary Information:**

The online version contains supplementary material available at 10.1038/s41598-024-82178-0.

## Introduction

Acute-on-chronic liver failure (ACLF) is a multifaceted syndrome characterized by a sudden deterioration of liver function accompanied by (multi-)organ failure in patients pre-existing liver disease^1–3^. The severity of ACLF is evidenced by a 28-day mortality ranging from 22% for patients with one organ failure (ACLF-1) to 77% for those with three or more organ failures (ACLF-3). Despite the advancements in critical care, these mortality rates remain alarmingly high^3–6^.

The prognosis of critically ill patients depends on the presence and severity of organ failures (OFs). However, ACLF patients face additional challenges due to specific clinical features of patients with advanced chronic liver disease, i.e. cirrhosis-associated immune dysfunction, which increases susceptibility for infections and sepsis^2,7,8^. Moreover, complications of portal hypertension such as gastrointestinal bleeding may worsen the prognosis and reduce ICU survival rates^9–12^.

An accurate risk stratification is crucial for ICU decision-making and optimizing patient care. Currently, ACLF is diagnosed based on the Chronic Liver Failure Consortium ACLF score (CLIF-C ACLFs) - the “gold-standard” for mortality risk stratification in patients with ACLF. While existing liver disease scores, i.e. the model for end-stage liver disease (MELD) and Child-Pugh score, reasonably predict overall mortality, they have not specifically been designed for ICU mortality prediction^13^. Efforts to refine the CLIF-C ACLFs predominantly rely on conventional adjustment methods, such as linear regression models, and may not fully capture the intricacies of the syndrome^9,14,15^. Machine learning (ML) approaches offer a promising avenue for improving outcome prediction in patients with ACLF by identifying hidden patterns within complex datasets, that conventional methods may overlook^16,17^. However, to date, no ML-based model is available to predict ICU mortality in patients with ACLF, representing an essential knowledge gap.

The primary objective of this study was to develop a ML-based model to more accurately predict ICU mortality in patients with ACLF, surpassing conventional scoring systems. By harnessing the power of ML algorithms, we aimed at unlocking hidden insights from patient data and identifying relevant patterns that could revolutionize ICU mortality prediction in this challenging patient cohort.

## Results

### Patients’ characteristics

206 retrospectively met the criteria for ACLF and were included in the analysis. Of those, 82 (40%) survived the ICU whereas 124 (60%) did not. The median age of all patients included was 56, ranging from 19 to 87 years (Table 1). Most patients were male (61%), with a median body mass index (BMI) of 28 kg/m², and 28% had preexisting diabetes mellitus type 2. At the time of study inclusion 17 patients (8%) had been diagnosed with hepatocellular carcinoma. Alcohol-associated liver disease was the primary etiology of advanced chronic liver disease (66%), with a significantly higher proportion among ICU non-survivors. Significant differences between ICU survivors and ICU non-survivors were furthermore noted in the white blood cell count (WBC, survivors 11.9/nl, non-survivors 14.9/nl, *p* = 0.004), C-reactive protein (CRP, survivors 467.62 nmol/l mg/l, non-survivors 586.67 nmol/l, *p* = 0.06), and interleukin-6 (IL-6, survivors 1067.5 pg/ml, non-survivors 6169.5 pg/ml, *p* = 0.007) (Supplementary Table 1). Liver function parameters, such as the total serum bilirubin level and INR were significantly worse in ICU non-survivors. Approximately 57% of patients had ACLF grade 3, with 73% in the non-survivor group compared to 33% in the survivor group. This corresponded with significantly worse respiratory parameters, such as the Horovitz quotient, and a higher necessity for hemodynamic support in the ICU non-survivors. The average ICU stay was approximately 12 days. 15 patients (7%) underwent liver transplant. The overall mortality rate was 72% (30% in ICU survivors) (Table 1). Additional baseline characteristics are presented in suppl. Table 1.

**Table 1:** Baseline characteristics of ICU survivors vs. non-survivors.

	All patients	ICU survivors	ICU non-survivors	*p* value
Patients, n (%)	206 (100)	82 (39.8)	124 (60.2)	
Characteristics				
Age [years], median (range)	56 (19–87)	54.8 (19–87)	56 (25–76)	0.596
Male gender, n (%)	125 (60.7)	50 (60.9)	75 (60.5)	0.944
HCC, yes (%)	17 (8.3)	8 (9.7)	9 (7.3)	0.524
BMI [kg/m^2^], median (range)	28 (15.6–62.5)	27.7 (17.3–59.5)	28.3 (15.6–62.5)	0.431
Etiology				
Alcohol, n (%)	135 (65.5)	46 (56.1)	89 (71.8)	0.023
Viral, n (%)	13 (6.3)	6 (7.3)	7 (5.6)	0.091
MASLD, n (%)	19 (9.2)	11 (13.4)	8 (6.4)	0.629
ACLF grade				
Grade 1, n (%)	25 (12.1)	18 (22.0)	7 (5.6)	< 0.0001
Grade 2, n (%)	64 (31.1)	37 (45.1)	27 (21.8)	< 0.0001
Grade 3, n (%)	117 (56.8)	27 (32.9)	90 (72.6)	< 0.0001
Type of decompensation				
Ascites, n (%)	178 (86.4)	69 (84.1)	109 (87.9)	0.441
Gastrointestinal hemorrhage, n (%)	48 (23.3)	24 (29.3)	24 (19.4)	0.099
Hepatic encephalopathy, n (%)	52 (25.2)	22 (26.8)	30 (24.2)	0.670
Sepsis, n (%)	115 (55.8)	33 (40.2)	82 (66.1)	< 0.0001
Laboratory parameters				
Bilirubin [µmol/l], median (range)	157.53 (4.62–655.58)	131.87 (4.62–655.58)	174.63 (7.18–632.67)	0.004
Creatinine [µmol/l], median (range)	235.15 (34.48–813.3)	222.77 (34.48–702.79)	245.76 (37.13–813.3)	0.339
INR, median (range)	2.2 (1.03–6.39)	1.85 (1.03–3.60)	2.42 (1.06–6.39)	< 0.0001
Blood gas analysis				
Lactate [mmol/l], median (range)	3.93 (0.6–18.0)	2.65 (0.6–12.1)	4.84 (0.6–18.0)	< 0.0001
ICU treatment data at admission				
Mechanical ventilation data				
Horovitz quotient [mmHg], median (range)	273.14 (49–623)	337.84 (94–623)	230.55 (49–519)	< 0.0001
FiO_2_ [%], median (range)	40 (21–100)	29 (21–100)	47 (21–100)	< 0.0001
Hemodynamic data				
Pharmacological hemodynamic support, yes, n (%)	163 (79.1)	52 (63.4)	111 (89.5)	< 0.0001
TPTD, yes, n (%)	37 (17.9)	4 (4.8)	33 (26.6)	0.0001
Length of ICU treatment				
ICU days, median (range)	12.1 (1-178)	13.2 (1–178)	11.4 (1-106)	0.262
Outcome				
OLT, yes, n (%)	15 (7.3)	10 (12.2)	5 (4.0)	0.1043
90-day mortality, n (%)	138 (66.9)	16 (19.5)	122 (98.4)	< 0.0001
Overall mortality, n (%)	149 (72.3)	26 (31.7)	124 (100.0)	0.0001
Survival [days], median (range)	328.3 (1–2285)	799.2 (5–2285)	13.4 (1–106)	< 0.0001

Baseline characteristics at the time point of ICU admission. For quantitative variables median and range (in parentheses) are given. Significance between ICU survivors and ICU non-survivors was assessed using the unpaired Mann-Whitney U or chi-squared test, respectively. Abbreviations: BMI, body mass index; MASLD, metabolic dysfunction-associated steatotic liver disease; INR, international normalized ratio; TPTD, transpulmonary thermodilution.

### Comparison of existing scores for ICU mortality prediction

**Fig. 1:**
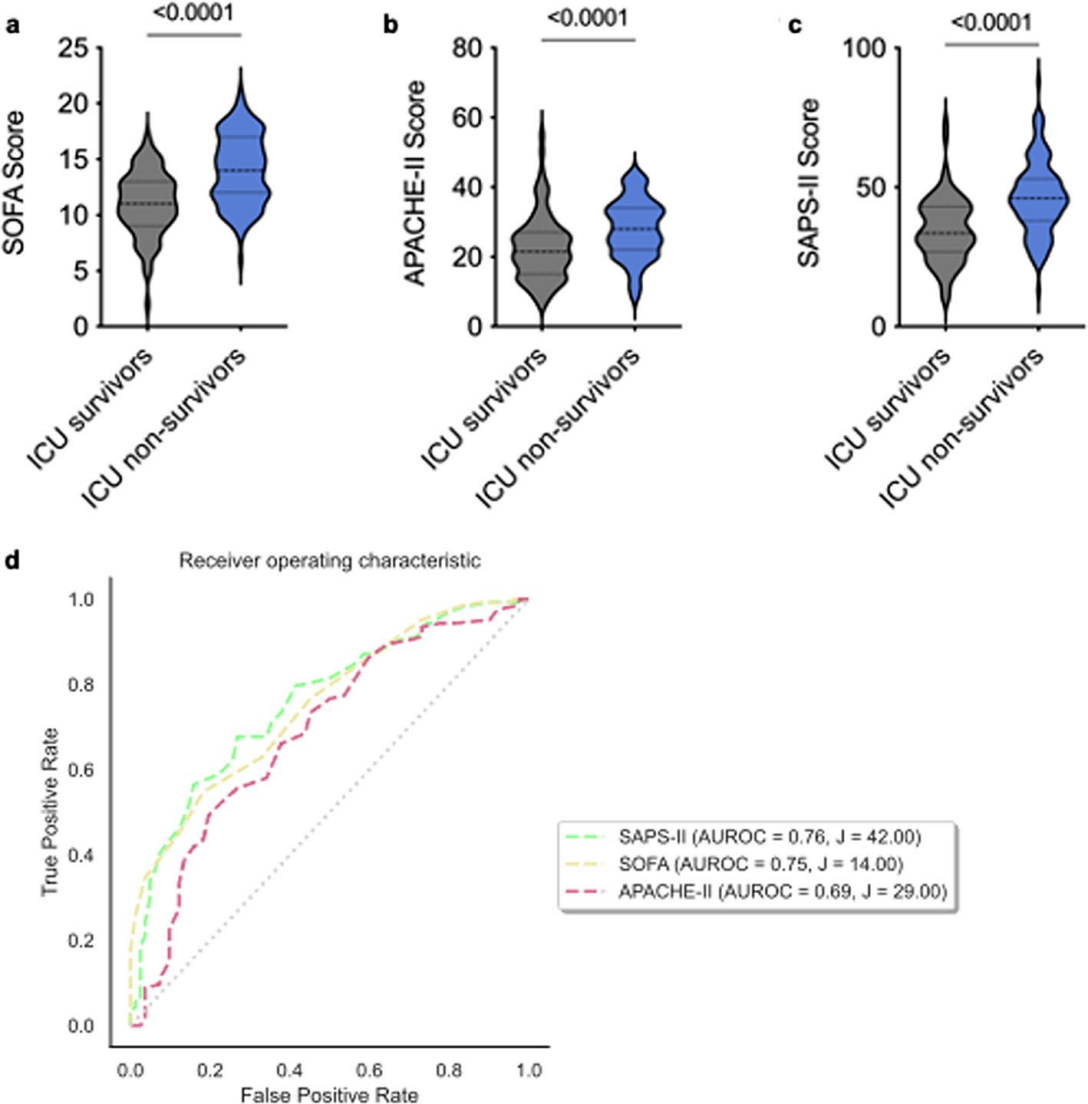
Comparison of existing scores for ICU mortality prediction. (**a**) Comparison of median SOFA scores between ICU survivors vs. ICU non-survivors, *p* < 0.0001. (**b**) Comparison of median APACHE-II scores between ICU survivors vs. ICU non-survivors, *p <* 0.0001. (**c**) Comparison of median SAPS-II scores between ICU survivors vs. ICU non-survivors, *p* < 0.0001. In (**a**–**c**) an unpaired two-tailed t-test was used. (**d**) Comparison of AUROC of scores estimating the ICU mortality in the whole cohort: SAPS-II (AUROC = 0.76; J = 42), SOFA (AUROC = 0.75, J = 14), APACHE-II (AUROC = 0.69, J = 29).

We observed significantly higher SOFA, APACHE-II and SAPS-II scores in ICU non-survivors compared to ICU survivors (each *p* < 0.0001; Fig. 1a–c). The best predictive capacity for ICU mortality was achieved with the SAPS-II score (AUROC 0.76, 95% CI: 0.47; 0.81) using an optimal cut-off of 42 determined by Youden´s index (Fig. 1d).

### Comparison of prognostic scores for mortality prediction in patients with advanced chronic liver disease

**Fig. 2:**
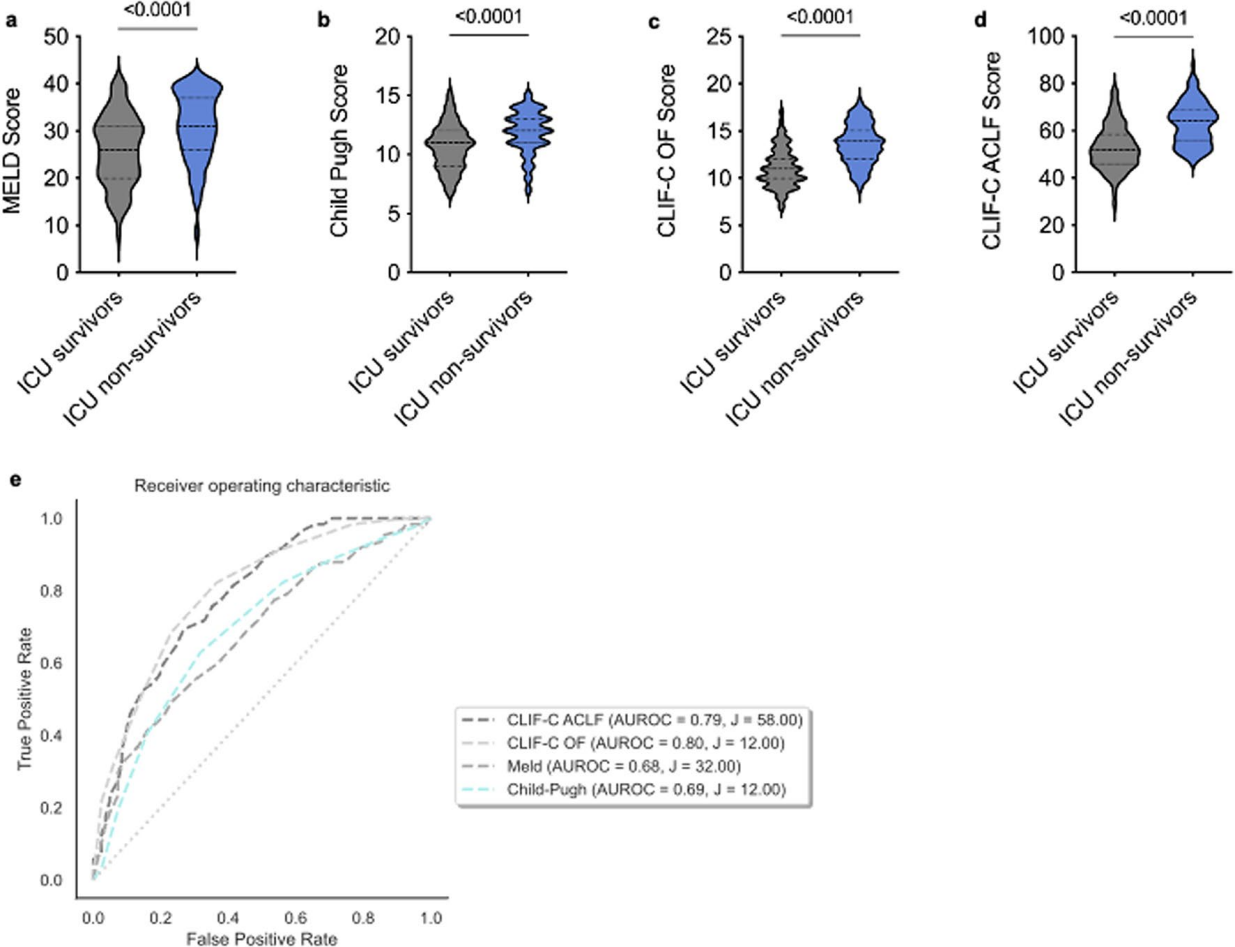
Comparison of existing scores for liver disease mortality prediction. Comparison of (**a**) MELD score, (**b**) Child-Pugh score, (**c**) CLIF-C OF score and (**d**) CLIF-C ACLF score between ICU survivors vs. ICU non-survivors. In (**a**–**d**) an unpaired, two-tailed t-test was used, each *p* < 0.0001. (**e**) Comparison of AUROC of scores estimating the mortality due to advanced liver disease in the whole cohort: CLIF-C ACLFs (AUROC = 0.79, J = 58), CLIF-C OF (AUROC, J = 12), MELD (AUROC = 0.68, J = 32), Child-Pugh (AUROC = 0.69, J = 12).

Given the mediocre predictive performance of the mentioned scores in this cohort, the performance of commonly applied scores for predicting overall mortality in patients with advanced liver disease was evaluated. ICU non-survivors had significantly higher MELD, Child-Pugh, CLIF-C OF and CLIF-C ACLF scores compared to ICU survivors (each *p* < 0.0001; Fig. 2a–d). The CLIF-C ACLFs had the best predictive capacity for ICU mortality (AUROC 0.80, 95% CI: 0.71; 0.99) with an optimal cut-off of 58 determined by Youden´s index (Fig. 2e).

### Establishment of the ACICU score

While established scoring systems exist for predicting overall mortality in patients with advanced liver diseases or ICU mortality in critically ill patients, there is currently no such system available for predicting ICU mortality especially in patients with ACLF. To address this gap, we utilized a ML-based model to develop an optimized scoring system for predicting ICU mortality.

We initially explored decision tree models as one of the ML-based approaches. Those models utilized 13 to 68 features and had only mediocre sensitivity (0.67–0.81) and unsatisfactory AUROC values (Suppl. Table S2). Consequently, we employed a logistic regression model, a form of supervised ML that identifies patterns in the training data to predict outcomes. This model calculates coefficients in a linear model to describe the relationship between a categorical dependent variable (ICU survival yes/no) and one or more independent variables^18^. The result is expressed as P(Y = 1), representing the probability of the outcome as a categorical value between 0 and 1.

The ML process generated seven logistic regression models using different combinations of features, ranging from five to thirteen (Suppl. Table S3). We observed that increasing the number of variables improved accuracy, aligning with findings by Harrel and his collaborators in 1996^19^. However, for clinical applicability, it was important to use the fewest possible number of variables. The logistic regression model with L1 regularization automatically selected the features without predetermined inputs. We evaluated each model’s performance using various metrics and selected the top-performing models for subsequent analysis.

**Fig. 3:**
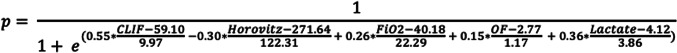
ACICU score formula.

Among the evaluated models, the newly defined ACICU score demonstrated the best performance metrics. This score employs five features: Horovitz quotient, FiO_2_, number of organ failures, lactate, and CLIF-C ACLFs (Fig. 3).

### Application and performance metrics of the ACICU score

The ACICU score ranges from 0 to 1, with higher values indicating a higher risk of ICU mortality. An exemplary calculation for a high- and low risk patient of our cohort is given in supplemental Table 4 (Suppl. Table S4).

**Fig. 4:**
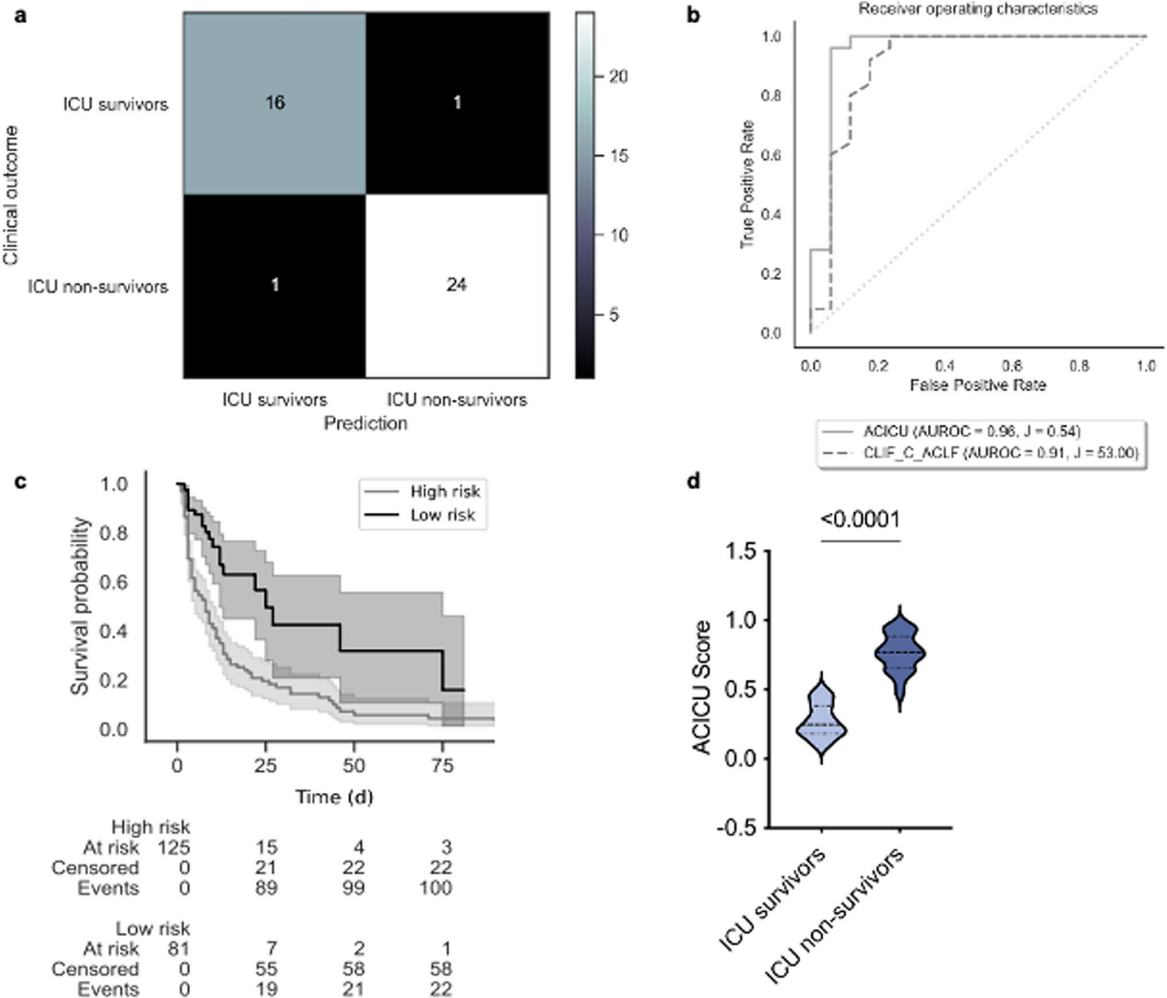
ACICU score validation. (**a**) Confusion matrix of the ACICU score in validation cohort. (**b**) Receiver operating characteristics of the ACICU score (AUROC = 0.96, J = 0.54) in comparison to CLIF-C ACLFs (AUROC = 0.91, J = 53) in validation cohort. (**c**) ICU survival days according to the ACICU score in whole cohort. High risk of ICU death was noted in patients with ACICU score above 0.54. (**d**) Comparison of the values of the ACICU score between ICU survivors vs. ICU non-survivors in whole cohort, unpaired, two-tailed t-test was used, *p* < 0.0001.

The confusion matrix demonstrated a low incidence of false negative and false positive results, achieving an overall performance of 0.95 (Fig. 4a; Table 2). Although the score’s calibration was inferior to other developed models, it was still deemed acceptable (Suppl. Table S3). The ACICU score achieved an average AUROC derived from 5-fold cross validation of 0.96 (95% CI: 0.87; 1.0) (Suppl. Fig. S2, Fig. 4b) compared to the CLIF-C ACLFs` AUROC of 0.91 (CI 95%: 0.88; 1.0) in the testing set (Fig. 4b). A cutoff of 0.54, determined by Youden´s index, allowed a clear differentiation of high risk and low risk for ICU mortality (Fig. 4c; Table 2). In our single-center cohort, the ACICU score was significantly higher in ICU non-survivors compared to ICU survivors (median: ICU survivors 0.27 vs. ICU non-survivors 0.77, Fig. 4d).

**Table 2:** Performance of the ACICU score.

	Target	ACICU score
Precision	1	0.95 (0.94–0.96)
Sensitivity	1	0.95 (0.94–0.96)
F1-score	1	0.95 (0.94–0.96)
Calibration-in-the-large	0.6	0.58
Calibration intercept	0	− 0.216
Calibration slope	1	2.7046
AUROC	1	0.96
Youden’s J		0.54
Number of used features		5
Features		‘d1_CLIF_C_ACLF’, ‘Number_OF’, ‘d1_Horovitz’, ‘d1_F_i_O_2_’, ‘d1_BGA_Lactate’
Coefficients		0.65130518
		0.14880095
		-0.29750186
		0.26440165
		0.35542168

Performance and calibration of the ACICU score in the validation cohort are described in the upper part of the table. X_n_ refers to the features, β_n_ refers to the coefficients as described in the formula for logistic regression in “Material and Methods” section.

The variables included in the ACICU score, especially Horovitz quotient and FiO_2_, emphasize respiratory failure. To assess the interrelationship among these variables, an intercorrelation analysis was conducted. This analysis revealed a strong positive correlation between the number of organ failures and CLIF-C ACLFs, while the Horovitz quotient and FiO_2_ exhibited a negative correlation. Only moderate correlations were observed for the other variables (Suppl. Table S5).

Patients who underwent OLT due to ACLF were classified as survivors during the establishment of the ACICU score, potentially confounding the results. However, a subgroup analysis excluding OLT patients still revealed an average AUROC of 0.86 for the ACICU score (Suppl. Fig. S2).

In addition to mortality prediction in ACLF patients we investigated whether the novel ACICU score could also support clinicians to decide which patient could especially benefit from OLT. However, the ACICU score did not show a difference between patients who benefited from liver transplant and those who did not (Suppl. Fig. S3).

### Futility analysis

**Fig. 5:**
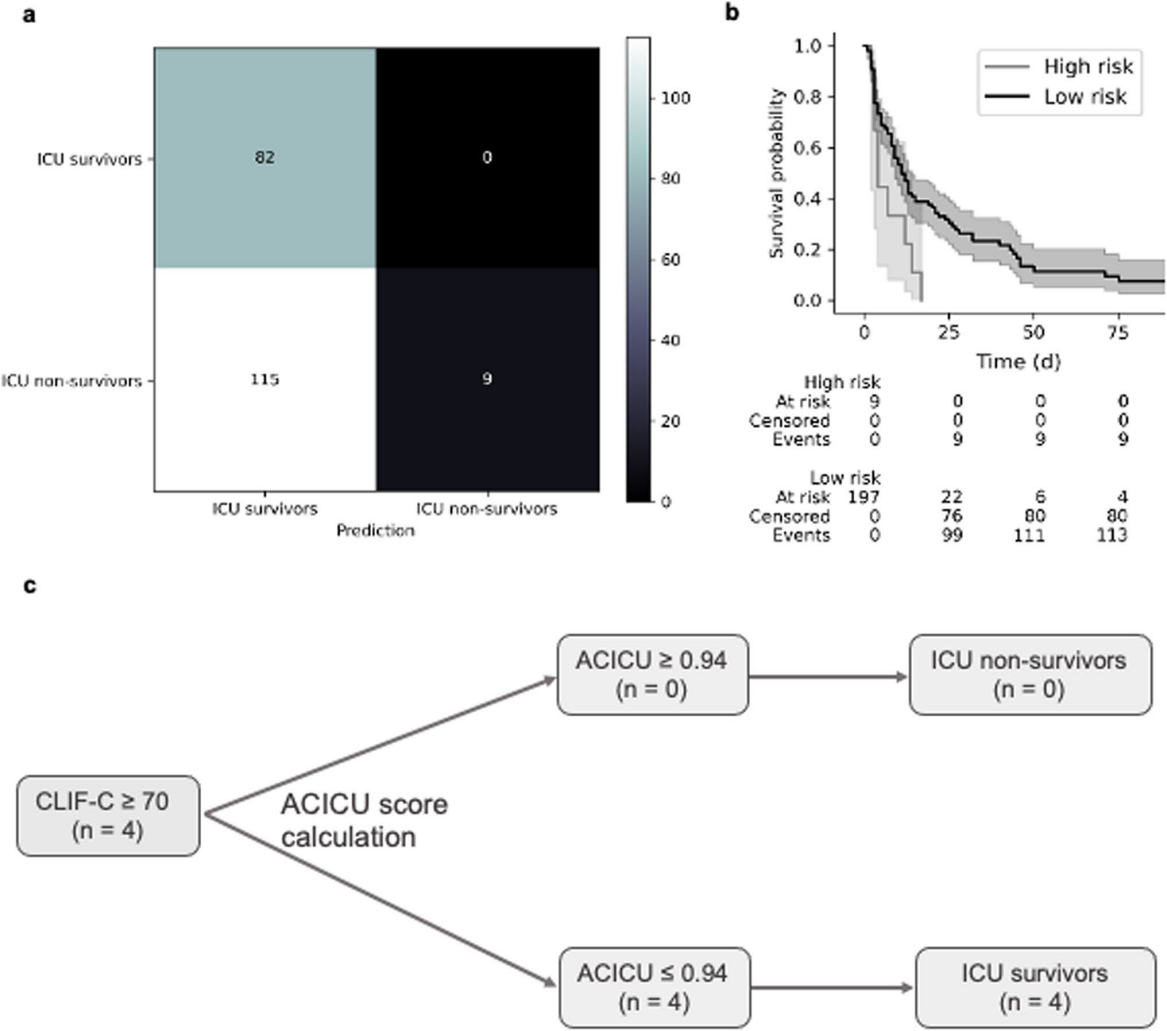
Futility analysis according to the ACICU score. ACICU score futility analysis was performed in the whole cohort. (**a**) Confusion matrix for the ACICU score with a threshold of 0.94 showing a 100% ICU mortality rate for patients above the threshold. (**b**) ICU survival probability according to the ACICU score. High risk of ICU death was noted in patients with an ACICU score above 0.94. (**c**) Flowchart outlining the sequence of score calculation to identify ACLF patients with a CLIF-C ACLFs of ≥ 70 who may still benefit from ICU treatment.

According to the ROC analysis, different thresholds for the ACICU score were assessed to predict ICU outcomes in critically ill patients with ACLF and identify those for whom prolonged ICU care might be futile. The highest specificity for determining ICU mortality was reached with an ACICU score ≥ 0.94 (Fig. 5a). Patients with an ACICU score below 0.94 had an ICU mortality rate of 58% (115 of 197), whereas patients above the threshold had a 100% mortality rate (Fig. 5b). Engelmann et al. defined a CLIF-C ACLFs cut-off above 70 as threshold for intensive care support for patients with ACLF^20^. To assess the value of the ACICU score as a dichotomized variable for identifying patients for whom prolonged ICU care might be futile, we compared the classification of patients using the Engelmann futility score cutoff^20^ and the ACICU score cutoff. ICU survivors were correctly classified in 78 (95.1%) cases by the CLIC-C ACLFs cutoff and in 82 (100%) by the ACICU score cutoff. ICU non-survivors were correctly classified in 29 (23.4%) by the CLIF-C ACLFs cutoff and in 9 (7.3%) cases by the ACICU score cutoff (Suppl. Table S6). This resulted in a positive predictive value of 0.23 using the CLIF-C ACLF and 0.07 using the ACICU score, and a negative predictive value of 0.95 and 1, respectively. In our cohort, 4 patients (12.1%) who had been classified as ICU non-survivors according to the CLIF-C ACLFs ≥ 70 did not decease. Of those, all 4 were correctly classified as ICU survivors according to the ACICU score (Fig. 5c). A suggested sequence of score calculation to identify patients with ACLF with a CLIF-C ACLFs of ≥ 70 who might still benefit from ICU treatment is outlined in Fig. 5c.

## Discussion

Recently, international consortia have developed models to predict short- and long-term mortality in patients with ACLF. However, these scores have not been specifically designed to predict ICU outcomes in patients with ACLF, limiting their utility in this context. We present the ACICU score – a novel, easy-to-use score derived from a ML-based algorithm using data from 206 critically ill patients with ACLF. Strikingly, the ACICU score was superior to the existing gold-standard CLIF-C ACLFs for prediction of ICU mortality in patients with ACLF and could therefore represent a valuable addition to existing prognostic tools.

The prognosis of critically ill patients, regardless of cirrhosis, is heavily influenced by the presence of organ failures, forming the basis for prognosis scores. The CLIF-C ACLFs represents the current gold standard for predicting overall mortality of patients with ACLF^13^. Consistent with previous findings, in our single center cohort the CLIF-C ACLFs outperformed other existing scores in predicting ICU mortality^13^. Recent efforts have aimed to improve the predictive accuracy of the CLIF-C ACLFs, especially for ICU mortality prediction as well as to evaluate futility of ICU treatment in patients with ACLF^20^. For instance, researchers have assigned higher weight to specific organ failures such as respiratory or circulatory failure, or even incorporated surrogate markers like the neutrophil–lymphocyte ratio (NLR)^9,14,15^.

However, all available studies in this context have made use of single conventional adjustment methods, e.g. logistic regression, that primarily aim at investigating relationships between variables^21^. We opted for logistic regression as basis for our predictive model as it directly estimates the probability of mortality within a specific time frame. This approach was preferable over time-to-event analysis for our study’s objective, which focused on evaluating the risk of death in ACLF patients during their ICU stay. Here, logistic regression provided a straightforward and interpretable measure of mortality risk, facilitating clinical decision-making and allowing futility assessment in this critical care setting. Moreover, the models based on logistic regression performed better than the miscellaneous evaluated models.

The ACICU score now presents the first model for ICU ACLF mortality prediction that is derived from an ML-based approach. ML algorithms are advantageous as they can learn from existing data thereby uncovering novel patterns^17^. There is evidence that ML in contrast to conventional analysis tools may especially improve prognosis prediction – for example, it was shown that ML-based approaches can improve mortality prediction in patients with acute coronary syndrome^22^. Notably, ML-based prediction models have also been developed in hepatological contexts such as for the prediction of hepatocellular carcinoma development as well as graft failure after liver transplantation^23,24^, suggesting the feasibility of ML-based models in hepatology.

In our study, 118 clinical and laboratory features were evaluated using ML to identify key differentiation criteria resulting in the ACICU score that incorporates the CLIF-C ACLFs, Horovitz quotient, FiO_2_, number of organ failures and lactate.

Both number of organ failures and lactate had previously been addressed as relevant prognostic parameters in patients with ACLF. For example, Cardoso et al. developed the LacOF model that combines lactate and number of organ failures and outperformed common ACLF mortality scores including the CLIF-C ACLF as well as CLIF-SOFA score^14^. The relevance of lactate in prediction of short-term mortality in critically ill patients with cirrhosis had previously been confirmed when Drolz et al. developed the so-called CLIF-C ACLFs_Lact_^25^.

9% of patients with ACLF present with pulmonary failure^3^. Already in the late 1980s, the need of mechanical ventilation was identified as one crucial prognostic factor of patients with liver cirrhosis requiring ICU treatment^26^. Subsequent studies confirmed that impaired oxygenation is a critical factor in predicting ICU mortality and could enhance the sensitivity of the ACICU score^9–12^. For example, Schulz et al. argued, that the grade of pulmonary impairment should be considered in the risk assessment of patients with ACLF and introduced the CLIF-C ACLF-R score. The fact, that our ML-based approach resulted in a score that combines three parameters that reflect pulmonary impairment – the CLIF-C ACLF itself, Horovitz quotient and FiO_2_ – further strengthens the relevance of respiratory failure in critically ill patients with ACLF.

In an intercorrelation analysis we specifically found a notable correlation between CLIF-C ACLFs and number of organ failures, as well as between FiO_2_ and Horovitz could be noted. While these correlations may raise concerns regarding multicollinearity and potentially weaken the model’s predictive performance, it is essential to note that the ACICU score still demonstrated robust predictive capability for ICU mortality. Future studies may explore methods to mitigate the impact of intercorrelations, such as feature selection techniques or regularization methods, to further enhance the model’s accuracy and reliability.

An ACICU score cutoff  ≥ 0.94 demonstrated 100% specificity in predicting mortality, meaning all patients surpassing this value died in the ICU. While the number of patients with ACICU ≥ 0.94 was modest (n = 9), our findings imply that in these patients, prolongation of ICU care might be futile and treatment options – including the need for palliative care – should be carefully evaluated. In contrast, 4 patients of our cohort were misclassified as ICU non-survivors by the Engelmann cutoff (CLIF-C ACLFs ≥ 70), but correctly classified as ICU survivors by the ACICU score^20^. Therefore, we suggest calculating the ACICU score after CLIF-C ACLFs determination to identify patients that were misclassified as non-survivors and still could profit from ICU therapy.

We acknowledge several limitations: First, the retrospective and single-center design of our study might limit the generalizability of our findings to a broader ACLF patient population. Although our study was internally validated with cross-sectional validation as advised for small data sets, larger multicenter studies are warranted to validate and extend the predictive capabilities of our ML-based model to diverse patient populations^27^. Second, although our study has yielded promising results, the high AUROC of our score may indicate potential overfitting. We have attempted to address this using fivefold cross-validation as well as lasso and ridge regression. Nevertheless, the generalizability of our score needs to be tested in external cohorts in future studies where hyperparameters can be fine-tuned to improve the robustness of our score. Third, as a single-center study, our findings might not fully represent the diversity of patient characteristics and clinical practices seen in multiple centers. Therefore, we recommend that future research should focus on conducting multi-center studies to assess the model’s performance across different healthcare settings and patient demographics.

In conclusion, our study offers valuable insights into the potential of a ML-based approach for predicting ICU mortality in patients with ACLF. The ACICU score achieved satisfactory performance and calibration metrics using readily available clinical parameters and outperformed the existing gold standard CLIC-C ACLFs. Overall, our findings represent a significant step forward in prognosis prediction in critically ill patients with ACLF.

The ACICU score can be calculated for scientific discussion using the ACICU score calculator available at www.acicu-score.com.

## Materials and methods

### Study design and patients’ characteristics

The study was conducted at the RWTH Aachen University Hospital. A total of *n* = 264 patients admitted to the medical ICU between August 2015 and May 2021 due to acute decompensation of liver cirrhosis were screened. Of those, *n* = 206 patients fulfilled the EF-CLIF ACLF criteria and were included in this retrospective analysis^3^. Exclusion criteria were patient´s age below 18 years, an anticipated ICU stay of less than 48 h, ICU admission due to acute poisoning, pregnancy or post-interventional observation after an elective procedure (such as portal vein embolization, transjugular intrahepatic portosystemic shunt insertion, or transarterial chemoembolization). The study protocol was approved by the local ethics committee (EK 150/06) of the University Hospital Aachen, RWTH Aachen University, Aachen, Germany, and conducted according to the ethical principles outlined both in the Declarations of Helsinki and Istanbul. Written informed consent was obtained from the patient, her/his spouse or legal guardian upon ICU admission.

### Score determination

Upon ICU admission, the MELD, Child-Pugh, CLIF-C ACLF and CLIF-C OF scores were calculated to assess severity of acute decompensation and ACLF. Clinical and laboratory parameters as well as the presence of hepatic encephalopathy according to the West Haven criteria^28^, the mean arterial pressure, use of vasopressors, partial arterial pressure of oxygen (paO_2_), FiO_2_, and necessity of mechanical ventilation were considered. The Sequential Organ Failure Assessment (SOFA), the Acute Physiology and Chronic Health Evaluation (APACHE) II and the Simplified Acute Physiology (SAPS) II scores were determined to evaluate the mortality risk of critically ill patients. For dynamic parameters, the worst value within the last 24 h was considered.

### Machine learning-based model

To predict ICU mortality, we explored both logistic regression and decision tree models.

In a logistic model, the log-odds of an event are modeled as a combination of one or more independent variables. Logistic regression estimates the coefficients in the linear combination, as exemplified by the SAPS score^29^. Decision tree models recursively split the dataset into subsets based on predictor variables and aim to maximize subset homogeneity with respect to the outcome variable^30^. Each node in the tree represents a decision based on a specific predictor variable, creating branches that lead to different outcomes. This process continues until a stopping criterion is met, such as a maximum tree depth or minimum samples per leaf node. Decision tree models and logistic regression models generate decision boundaries differently. Decision tree models bisect the data into smaller regions, though they are prone to overfitting^31^. In contrast, logistic regression fits a single line to separate the data into two regions, which may limit its flexibility.

The ML process started with data preparation for training and testing. Categorical features were converted into binary classifications. Features with more than 20 percent missing data were excluded, resulting in 118 features. The data set was stratified and split into training (164 patients, 80%) and testing sets (42, 20%). Missing data were addressed using k-nearest neighbor imputation with ten neighbors, and the data were standardized using the standard scaler.

Model development consisted of two phases: In the training phase, the model was trained to predict ICU mortality using the training data set. Hyperparameters were optimized using a grid search with fivefold cross-validation. Both Lasso regression (L1 regularization) and Ridge regression (L2 regularization) were applied to select features and address overfitting, respectively. Models were only trained to the training set to avoid information bleed-through. In the testing phase, the model’s performance on the testing data set was evaluated using fivefold cross-validation. Performance metrics included precision, sensitivity, and the F1-Score. Model calibration (prediction of the absolute risk^32^) and discrimination (differentiation of high and low-risk groups^32^) were assessed using calibration-in-the-large, calibration slope and intercept, AUROC and Youden’s index.

For model development, the scikit-learn package (version 1.3.0) for Python was used, while the lifelines package (version 0.27.3) was applied for survival analysis^33,34^. Logistic regression was employed with the following standardized formula:

$$\:{\rm\:P}\left(Yi=1\right)\:=\:\frac{{e}^{(\beta\:0+\beta\:1\chi\:1+\dots\:+\beta\:n\chi\:n)}}{1+{e}^{(\beta\:0+\beta\:1\chi\:1+\dots\:+\beta\:n\chi\:n)}}$$

X1, X2, X3 … represent the features, β1, β2, β3, … are the coefficients of the formula and reflect how the features poured into the computation. Features were standardized by their means and standard deviations, and nominal features were transformed into binary features.

### Statistical analysis

Statistical analysis was performed using SPSS (Version 26, SPSS Inc., Chicago, USA) and Python (Version 3.9.7, Python Software Foundation, Beaverton, USA). Data visualization was conducted with GraphPad Prism 6.0 (GraphPad Software, La Jolla, USA). Outliers were identified using the Grubbs’ test, and normal distribution was assessed with the Shapiro-Wilk test. The Mann-Whitney U test was used for unpaired samples with more than two groups, and the Chi-square test was applied to nominal variables. The Youden index determined optimal cut-off values for prognostic parameters. AUROC statistics were calculated by plotting the true positive versus false positive rate. Kaplan-Meier curves depicted patient survival. A p-value of less than 0.05 was considered statistically significant.

## Electronic supplementary material

Below is the link to the electronic supplementary material.

Supplementary Material 1

## Data Availability

The data that support the findings of this study are available from the corresponding author upon reasonable request. Some data may not be available because of privacy or ethical reasons.

## Abbreviations

ACLF: Acute-on-chronic liver failure
ACICU: Aachen ACLF ICU score
APACHE II: Acute physiology and chronic health evaluation II
CLIF-C ACLFs: Chronic liver failure consortium (CLIF-C) ACLF score
CLIF-C OFs: Chronic liver failure consortium (CLIF-C) organ failure score
ICU: Intensive care unit
INR: International normalized ratio
L1: Lasso regression
L2: Ridge regression
MELD: Model of end-stage liver disease
ML: Machine learning
OF: Organ failures
SAPS II: Simplified acute physiology score II
SOFA II: Sequential organ failure assessment II score

## References

[1] Arroyo, V., Moreau, R., Jalan, R., Gines, P. & Study, E.-C. C. C. Acute-on-chronic liver failure: A new syndrome that will re-classify cirrhosis. *J. Hepatol.* **62**, S131-143. 10.1016/j.jhep.2014.11.045 (2015). 25920082 10.1016/j.jhep.2014.11.045

[2] Arroyo, V., Moreau, R. & Jalan, R. Acute-on-chronic liver failure. *N. Engl. J. Med.* **382**, 2137–2145. 10.1056/NEJMra1914900 (2020). 32459924 10.1056/NEJMra1914900

[3] Moreau, R. et al. Acute-on-chronic liver failure is a distinct syndrome that develops in patients with acute decompensation of cirrhosis. *Gastroenterology* **144**, 1426–1437. 10.1053/j.gastro.2013.02.042 (2013). 23474284 10.1053/j.gastro.2013.02.042

[4] Gustot, T. et al. Clinical course of acute-on-chronic liver failure syndrome and effects on prognosis. *Hepatology* **62**, 243–252. 10.1002/hep.27849 (2015). 25877702 10.1002/hep.27849

[5] Galbois, A. et al. Improved prognosis of septic shock in patients with cirrhosis: A multicenter study. *Crit. Care Med.* **42**, 1666–1675. 10.1097/CCM.0000000000000321 (2014). 24732239 10.1097/CCM.0000000000000321

[6] McPhail, M. J. W. et al. Incidence and outcomes for patients with cirrhosis admitted to the united kingdom critical care units. *Crit. Care Med.* **46**, 705–712. 10.1097/CCM.0000000000002961 (2018). 29309369 10.1097/CCM.0000000000002961 PMC5899891

[7] Moreau, R. et al. Blood metabolomics uncovers inflammation-associated mitochondrial dysfunction as a potential mechanism underlying ACLF. *J Hepatol* **72**, 688–701. 10.1016/j.jhep.2019.11.009 (2020). 31778751 10.1016/j.jhep.2019.11.009

[8] Bruns, T., Zimmermann, H. W. & Stallmach, A. Risk factors and outcome of bacterial infections in cirrhosis. *World J. Gastroenterol.* **20**, 2542–2554. 10.3748/wjg.v20.i10.2542 (2014). 24627590 10.3748/wjg.v20.i10.2542 PMC3949263

[9] Schulz, M. S. et al. Pulmonary impairment independently determines mortality in critically ill patients with acute-on-chronic liver failure. *Liver Int.*10.1111/liv.15343 (2022). 35727853 10.1111/liv.15343

[10] Levesque, E. et al. Prospective evaluation of the prognostic scores for cirrhotic patients admitted to an intensive care unit. *J. Hepatol.* **56**, 95–102. 10.1016/j.jhep.2011.06.024 (2012). 21835136 10.1016/j.jhep.2011.06.024

[11] Levesque, E., Saliba, F., Ichai, P. & Samuel, D. Outcome of patients with cirrhosis requiring mechanical ventilation in ICU. *J. Hepatol.* **60**, 570–578. 10.1016/j.jhep.2013.11.012 (2014). 24280294 10.1016/j.jhep.2013.11.012

[12] Rabe, C. et al. Does intubation really equal death in cirrhotic patients? Factors influencing outcome in patients with liver cirrhosis requiring mechanical ventilation. *Intensive Care Med.* **30**, 1564–1571. 10.1007/s00134-004-2346-x (2004). 15292984 10.1007/s00134-004-2346-x

[13] Jalan, R. et al. Development and validation of a prognostic score to predict mortality in patients with acute-on-chronic liver failure. *J. Hepatol.* **61**, 1038–1047. 10.1016/j.jhep.2014.06.012 (2014). 24950482 10.1016/j.jhep.2014.06.012

[14] Cardoso, F. S. et al. Lactate and number of organ failures predict intensive care unit mortality in patients with acute-on-chronic liver failure. *Liver Int.* **39**, 1271–1280. 10.1111/liv.14083 (2019). 30825255 10.1111/liv.14083

[15] Chiriac, S. et al. Prognostic value of neutrophil-to-lymphocyte ratio in cirrhotic patients with acute-on-chronic liver failure. *Turk. J. Gastroenterol.* **31**, 868–876. 10.5152/tjg.2020.19838 (2020). 33625999 10.5152/tjg.2020.19838 PMC7928244

[16] Obermeyer, Z. & Emanuel, E. J. Predicting the future - big data, machine learning, and clinical medicine. *N. Engl. J. Med.* **375**, 1216–1219. 10.1056/NEJMp1606181 (2016). 27682033 10.1056/NEJMp1606181 PMC5070532

[17] Spann, A. et al. Applying machine learning in liver disease and transplantation: A comprehensive review. *Hepatology* **71**, 1093–1105. 10.1002/hep.31103 (2020). 31907954 10.1002/hep.31103

[18] Cox, D. R. The regression analysis of binary sequences. *J. R. Stat. Soc.: Ser. B (Methodol.)* **20**, 215–232. 10.1111/j.2517-6161.1958.tb00292.x (1958).

[19] Harrell, F. E. Jr., Lee, K. L. & Mark, D. B. Multivariable prognostic models: Issues in developing models, evaluating assumptions and adequacy, and measuring and reducing errors. *Stat. Med.* **15**, 361–387. 10.1002/(sici)1097-0258(19960229)15:4%3c361::Aid-sim168%3e3.0.Co;2-4 (1996). 8668867 10.1002/(SICI)1097-0258(19960229)15:4<361::AID-SIM168>3.0.CO;2-4

[20] Engelmann, C. et al. Validation of CLIF-C ACLF score to define a threshold for futility of intensive care support for patients with acute-on-chronic liver failure. *Crit. Care* **22**, 254. 10.1186/s13054-018-2156-0 (2018). 30305132 10.1186/s13054-018-2156-0 PMC6180662

[21] Rajula, H. S. R., Verlato, G., Manchia, M., Antonucci, N. & Fanos, V. Comparison of Conventional Statistical Methods with Machine Learning in Medicine: Diagnosis, Drug Development, and Treatment. *Medicina (Kaunas)* **56**, 10.3390/medicina56090455 (2020). 10.3390/medicina56090455 PMC7560135 32911665

[22] Hernesniemi, J. A. et al. Extensive phenotype data and machine learning in prediction of mortality in acute coronary syndrome - the MADDEC study. *Ann. Med.* **51**, 156–163. 10.1080/07853890.2019.1596302 (2019). 31030570 10.1080/07853890.2019.1596302 PMC7857486

[23] Singal, A. G. et al. Machine learning algorithms outperform conventional regression models in predicting development of hepatocellular carcinoma. *Am. J. Gastroenterol.* **108**, 1723–1730. 10.1038/ajg.2013.332 (2013). 24169273 10.1038/ajg.2013.332 PMC4610387

[24] Lau, L. et al. Machine-learning algorithms predict graft failure after liver transplantation. *Transplantation* **101**, e125–e132. 10.1097/tp.0000000000001600 (2017). 27941428 10.1097/TP.0000000000001600 PMC7228574

[25] Drolz, A. et al. Lactate improves prediction of short-term mortality in critically ill patients with cirrhosis: A multinational study. *Hepatology* **69**, 258–269. 10.1002/hep.30151 (2019). 30070381 10.1002/hep.30151

[26] Shellman, R. G., Fulkerson, W. J., DeLong, E. & Piantadosi, C. A. Prognosis of patients with cirrhosis and chronic liver disease admitted to the medical intensive care unit. *Crit. Care Med.* **16**, 671–678. 10.1097/00003246-198807000-00005 (1988). 3371043 10.1097/00003246-198807000-00005

[27] Eertink, J. J. et al. External validation: A simulation study to compare cross-validation versus holdout or external testing to assess the performance of clinical prediction models using PET data from DLBCL patients. *EJNMMI Res.* **12**, 58. 10.1186/s13550-022-00931-w (2022). 36089634 10.1186/s13550-022-00931-w PMC9464671

[28] Conn, H. O. et al. Comparison of lactulose and neomycin in the treatment of chronic portal-systemic encephalopathy A double blind controlled trial. *Gastroenterology* **72**, 573–583 (1977). 14049

[29] Le Gall, J. R., Lemeshow, S. & Saulnier, F. A new simplified acute physiology score (SAPS II) based on a European/North American multicenter study. *Jama* **270**, 2957–2963. 10.1001/jama.270.24.2957 (1993). 8254858 10.1001/jama.270.24.2957

[30] Rokach, L. M., Oded. Top-down induction of decision trees classifiers - a survey. *IEEE Transactions on Systems, Man, and Cybernetics, Part C (Applications and Reviews)* **35**, 476–487. 10.1109/TSMCC.2004.843247 (2005).

[31] Khoshgoftaar, T. M. & Allen, E. B. Controlling overfitting in classification-tree models of software quality. *Emp. Softw. Eng.* **6**, 59–79. 10.1023/A:1009803004576 (2001).

[32] Pencina, M. J., D'Agostino, R. B., Sr., D'Agostino, R. B., Jr. & Vasan, R. S. Evaluating the added predictive ability of a new marker: from area under the ROC curve to reclassification and beyond. *Stat. Med.* **27**, 157–172; discussion 207–112. 10.1002/sim.2929 (2008). 10.1002/sim.2929 17569110

[33] Pedregosa, F. et al. Scikit-learn: Machine learning in python. *J. Mach. Learn. Res.* **12**, 2825–2830 (2011).

[34] Davidson-Pilon, C. Lifelines: Survival analysis in python. *Journal of Open Source Software* **40**, 10.21105/joss.01317 (2019).

