# Supplementary material for: The Aachen ACLF ICU score predicts ICU mortality in critically ill patients with acute-on-chronic liver failure
Source: Sci Rep. 2024 Dec 16;14:30497. doi: 10.1038/s41598-024-82178-0 (PMC11649908; doi:10.1038/s41598-024-82178-0)
Supplement: Supplementary file 1 — Supplementary Material 1 [file 41598_2024_82178_MOESM1_ESM.pdf]

Supplementary

The Aachen ACLF ICU score predicts ICU mortality in critically ill patients with acute-on-chronic liver failure

Maike Rebecca Pollmanns[1], Bastian Kister[2], Samira Abu Jhaisha[1], Jule K. Adams[1], Elena Kabak[1], Jonathan Frederik Brozat[1, 3], Carolin Victoria Schneider[1], Philipp Hohlstein[1], Tony Bruns[1], Lars Küpfer[2], Christian Trautwein[1, 4], Alexander Koch[1,*], Theresa Hildegard Wirtz[1,*]

<u>Table of contents</u>

Table S1: Additional baseline characteristics of ICU survivors vs. ICU non-survivors
Table S2: Comparison of different decision tree models with logistic regression models
Table S3: Comparison of the performance of the generated logistic regression models
Table S4: Calculation of ACICU score in two exemplary patients
Table S5: Correlation analysis of the features of the ACICU Score
Table S6: Comparison of ACICU and CLIF-C ACLF score
Figure S1: ACICU Score validation
Figure S2: ACICU Score in ACLF patients not receiving liver transplantation
Figure S3: ACICU Score in ACLF patients receiving liver transplantation

Suppl. Table S1: Additional baseline characteristics of ICU survivors vs. ICU non-survivors

	**All Patients**	**ICU survivors**	**ICU non-survivors**	***p*-Value**
**Characteristics**				
Diabetes mellitus, yes (%)	55 (27)	26 (32)	29 (24)	0.186
**Laboratory parameters**				
WBC [n/nl], median (range)	13.7 (0.9 – 49.9)	11.94 (2.6 – 49.9)	14.9 (0.9 – 41.9)	0.004
Hb [g/dl], median (range)	8.97 (1.5 – 17.5)	8.94 (3.9 – 16.15)	8.98 (1.5 – 17.5)	0.643
PLT [n/nl], median (range)	94.13 (7.0 – 394.0)	101.7 (21 – 386)	89.1 (7 – 394)	0.166
AT III (%), median (range)	22.48 (2.0 – 65)	28.6 (4 – 59) n=41	20.2 (2 – 65) n=41	0.099
Fibrinogen [mg/dl], median (range)	138.46 (21.0 – 538.0)	132.2 (46 – 253)	140.7 (21 – 538)	0.823

eGFR [ml/min/1,73m²], median (range)	40.0 (4.4 – 163.3)	45.9 (6.0 – 163.3)	36.05 (4.4 – 130.1)	0.240
AST [U/l], median (range)	653.3 (13 – 17398)	393.3 (13 – 16294)	827.6 (13 – 17398)	< 0.0001
ALT [U/l], median (range)	264.43 (5 – 6886)	186.38 (7 – 6886)	316.47 (5 – 6030)	0.021
Albumin [g/dl], median (range)	2.77 (1.10 – 5.10)	2.95 (1.70 – 5.10)	2.65 (1.1 – 4.7)	0.009
Ammonia [µmol/l], median (range)	88.7 (10 – 418)	78.6 (10 – 328)	94.8 (10 – 418)	0.078
LDH [U/l], median (range)	630.7 (68 – 12194)	448.5 (82 – 11585)	753.1 (68 – 12194)	0.004
Na^+ [mmol/l], median (range)	137 (108 – 163)	137 (123 – 156)	137 (108 – 163)	0.906
K^+ [mmol/l], median (range)	4.53 (2.7 – 7.7)	4.53 (3.1 – 7.7)	4.52 (2.7 – 6.6)	0.722
BNP [pg/ml], median (range)	6192.64 (8.3 – 43935.0)	6107.36 (25.3 – 43935.0)	6248.01 (8.3 – 42757.0)	0.048
CRP [mg/l], median (range)	56.6 (1.7 – 245.2)	49.1 (2.4 – 244.6)	61.6 (1.7 – 245.2)	0.060
PCT [ng/ml], median (range)	4.38 (0.07 – 64.42)	4.71 (0.07 – 63.18)	4.17 (0.09 – 64.42)	0.025
Il-6 [pg/ml], median (range)	4108 (9.6 – 136014.0)	1067.5 (9.6 – 15234.0)	6168.5 (20.63 – 136014.0)	0.007
**Reason for decompensation of cirrhosis**				
Bleeding, n (%)	48 (23)	24 (30)	24 (20)	0.099
Sepsis, n (%)	115 (56)	33 (40)	82 (66)	< 0.0001
SBP, n (%)	44 (22)	13 (16)	31 (25)	0.282
pulmonary, n (%)	47 (23)	10 (12)	37 (30)	0.003
**ICU Data at the day of admission**				
Ventilation Data				
mechanical ventilation, yes (%)	101 (49)	23 (28)	78 (63)	0.0001
RF [n/min], median (range)	19 (7 – 55)	18 (7- 29)	20 (11 – 33)	0.024
p_aO_2 [mmHg], median (range)	87 (42 – 156)	89 (42 – 156)	85 (49 – 146)	0.421
PEEP [cm H_2O], median (range)	8.5 (5 – 15)	8.1 (5 – 15)	8.57 (5 – 15)	0.309
P_{insp} [mbar], median (range)	22 (8 – 32)	19 (8 – 30)	23 (12 – 32)	0.007
Renal replacement data				
Renal replacement therapy, yes (%)	87 (42)	24 (30)	63 (51)	0.002

Baseline patient characteristics at the time point of admission to ICU. For quantitative variables median and range (in parentheses) are given. *Abbreviations are*: WBC, white blood cell count; Hb, hemoglobin; PLT, platelet; AT III, antithrombin III; GFR,

glomerular filtration rate; AST, aspartate aminotransferase; ALT, alanine transaminase; LDH, lactic acid dehydrogenase; BNP, brain natriuretic peptide; CRP, C-reactive protein; PCT, procalcitonin; IL-6, interleukin-6; SBP, spontaneous bacterial peritonitis; RF, respiratory frequency; PEEP, positive end-expiratory pressure.

Suppl. Table S2: Comparison of different decision tree models with logistic regression models

	**Target**	**CLIF – L1 - L2**	**Tree 1**	**Tree 2**	**Tree 3**	**Tree 4**
**Input features**			all	R+C	All + CLIF-C ACLF	Model 6/7
**Precision**	1	0.91 (0.84-0.96)	0.66 (0.60-0.70)	0.81 (0.85-0.79)	0.74 (0.69-0.77)	0.67 (0.59-0.72)
**Sensitivity**	1	0.90 (0.94-0.88)	0.67 (0.53-0.76)	0.81 (0.65-0.92)	0.74 (0.65-0.80)	0.67 (0.59-0.72)
**F1-score**	1	0.91 (0.89-0.92)	0.66 (0.56-0.73)	0.80 (0.73-0.85)	0.74 (0.67-0.78)	0.67 (0.59-0.72)
**Calibration-in-the-large**	0.6	0.5	0.64	0.67	0.62	0.61
**AUROC**	1	0.95	0.64	0.8	0.71	0.67
**Youden's J**		0.50	1.00	0.75	1.00	0.25
**Number of used features**		13	67	16	68	13

Evaluated tree models in comparison to the first logistic regression model. In the first step all features were evaluated, in the second step features related to organ failures were tested. *Abbreviations are*: CLIF, Chronic Liver Failure Consortium (CLIF-C) ACLF score; NOF, number of organ failures; L1, Lasso Regression; L2, Ridge Regression.

Suppl. Table S3: Comparison of the performance of the generated logistic regression models. Input features were related to organ failures, addition of the number of organ failures (NOF) was also tested.

**Model number**		**Model 1**	**Model 2**	**Model 3**	**Model 4**	**Model 5 (ACICU Score)**	**Model 6**	**Model 7**
**Regularization**		L1	L1 -> L2	L1	L2	L2	L1	L2
**Precision**	1	0.84 (0.75 – 0.91)	0.9 (0.88 – 0.92)	0.9 (0.88 – 0.92)	0.9 (0.88 – 0.92)	0.95 (0.94 – 0.96)	0.89 (0.80 – 0.95)	0.88 (0.76 – 0.95)
**Sensitivity**	1	0.83 (0.80 – 0.88)	0.9 (0.88 – 0.92)	0.9 (0.88 – 0.92)	0.9 (0.88 – 0.92)	0.95 (0.94 – 0.96)	0.88 (0.84 – 0.94)	0.86 (0.94 – 0.80)
**F1-score**	1	0.83 (0.81 – 0.85)	0.9 (0.88 – 0.92)	0.9 (0.88 – 0.92)	0.9 (0.88 – 0.92)	0.95 (0.94 – 0.96)	0.88 (0.86 – 0.89)	0.86 (0.84 – 0.87)
**Calibration-in-the-large**	0.6	0.57	0.6	0.5393	0.5952	0.58	0.535	0.50
**Calibration intercept**	0	0.03	- 0.04	0.2192	- 0.04	- 0.216	0.56	0.8833
**Calibration slope**	1	3.22	1.81	3.7	1.8095	2.7046	1.59	243.32
**AUROC**	1	0.93	0.95	0.94	0.95	0.96	0.91	0.95
**Youden's J**		0.49	0.47	0.53	0.47	0.54	0.41	0.50
**Number of used features**		8	5	5	5	5	13	13
**Features**		d1_Horowitz', 'd1_FiO2', 'd1_paO2', 'd1_Ventilation',	'd1_CLIF_C_ACLF', 'd1_Horowitz', 'd1_FiO2',	'd1_CLIF_C_ACLF', 'd1_Horowitz', 'd1_FiO2',	d1_CLIF_C_ACLF', 'd1_Horowitz', 'd1_FiO2', 'd1_TPTD,	'd1_CLIF_C_ACLF', 'Number_OF', 'd1_Horowitz',	'Number_OF', 'd1_AF', 'd1_Horowitz', 'd1_FiO2', 'd1_paO2', 'd1_Ventilation', 'cause of sepsis, 'd1_MAP',	Number_OF', 'd1_AF', 'd1_Horowitz', 'd1_FiO2', 'd1_paO2',

		'd1_MAP', 'd1_TPTD, 'd1_RR_Sys' , 'd1_Lactate'	'd1_TPTD, 'd1_ Lactate'	'd1_TPTD, 'd1_ Lactate'	'd1 _Lactate'	'd1_FiO2', 'd1_ Lactate’	'd1_vasopressors','d1_ TPTD, 'd1_heart rate', 'd1_RR_Sys', 'd1_ Lactate'	'd1_Ventilation', cause of sepsis', 'd1_MAP', 'd1_vasopressos', 'd1_TPTD, 'd1_heart rate', 'd1_RR_Sys', 'd1_ Lactate'
**Coefficients**		-0.3472606<br>0.21422031<br>-0.03058471<br>0.07092862<br>-0.16473165<br>0.25087789<br>-0.05219007<br>0.2849164	0.7937202<br>-0.30231107<br>0.28361903<br>0.44091147<br>0.29946303	0.49873336<br>-0.21536315<br>0.06984075<br>0.08288953<br>0.05421657	0.7937202<br>-0.30231107<br>0.28361903<br>0.44091147<br>0.29946303	0.65130518<br>0.14880095<br>-0.29750186<br>0.26440165<br>0.35542168	0.42208604<br>0.02775644<br>0.23321895<br>0.82631475<br>-0.5261933<br>0.3846778<br>0.05338636<br>-0.3745731<br>-0.20863558<br>0.37922956<br>0.08786658<br>-0.09197543<br>0.40577534	0. 00247497<br>0.00097648<br>-0.00284844<br>0.00274555<br>-0.00052981<br>0.00226619<br>0.00126243<br>-0.00120199<br>0.00181572<br>0.00190038<br>0.00104307<br>-0.00096393<br>0.00204423

Used formula for logistic regression as described in Material and Methods. Performance and calibration of the validation cohort are described in the upper part of the table. X_n refers to the features, β_n refers to the coefficients. *Abbreviations are:* CLIF, Chronic Liver

Failure Consortium (CLIF-C) ACLF score; NOF, number of organ failures; L1, Lasso Regression; L2, Ridge Regression; TPTD, Transpulmonary Thermodilution

Suppl. Table S4: Calculation of ACICU score in two exemplary patients

	**Patient 1**	**Patient 2**
**CLIF-C ACLFs**	52	59
**Horovitz [mmHg]**	485	168
**FiO_2 [%]**	21	70
**Number of Organ Failures**	2	3
**Lactate [mmol/l]**	0.9	8.5
**ACICU score**	0.2	0.8
**Risk group**	Low risk	High risk
**Estimated risk of ICU mortality [%]**	24	82

Patient 1 representing a low risk patient, patient 2 representing a high risk patient with ACLF. *Abbreviations are:* CLIF-C ACLFs, Chronic Liver Failure Consortium (CLIF-C) ACLF score.

Suppl. Table S5: Correlation analysis of the features of the ACICU score

	**CLIF-C ACLF**	**NOF**	**Horovitz**	**FiO2**	**Lactate**
**CLIF-C ACLF**	1.000000	0.618330	- 0.375265	0.367614	0.316619
**NOF**	0.618330	1.000000	- 0.354321	0.359048	0.242783
**Horovitz**	- 0.375265	- 0.354321	1.000000	- 0.822832	- 0.255031
**FiO2**	0.367614	0.359048	- 0.822832	1.000000	0.339996
**Lactate**	0.316619	0.242783	- 0.255031	0.339996	1.000000

Correlation analysis showing pairwise the Pearson coefficients. *Abbreviations are:* CLIF-C ACLF: CLIF, Chronic Liver Failure Consortium (CLIF-C) ACLF score; NOF, number of organ failures

Suppl. Table S6: Comparison of ACICU and CLIF-C ACLF score

		**ICU survivors (n=82) (%)**	**ICU non-survivors (n=124) (%)**
**CLIF-C ACLF ≥ 70**	Yes (%)	4 (12.1)	**29 (23.4)**
	No (%)	**78 (95.1)**	95 (76.6)
**ACICU ≥ 0.94**	Yes (%)	0 (0)	**9 (7.3)**
	No (%)	**82 (100)**	115 (92.7)

Analysis of performance metrics of true and false classification of patients who do or do not survive ICU by the respective thresholds of CLIF-C ACLFs of 70 and ACICU score of 0.94. Percentages in the parentheses refer to the true survival status; number and percentage of truly classified patients are in bold.

Suppl. Figure S1: ACICU score validation

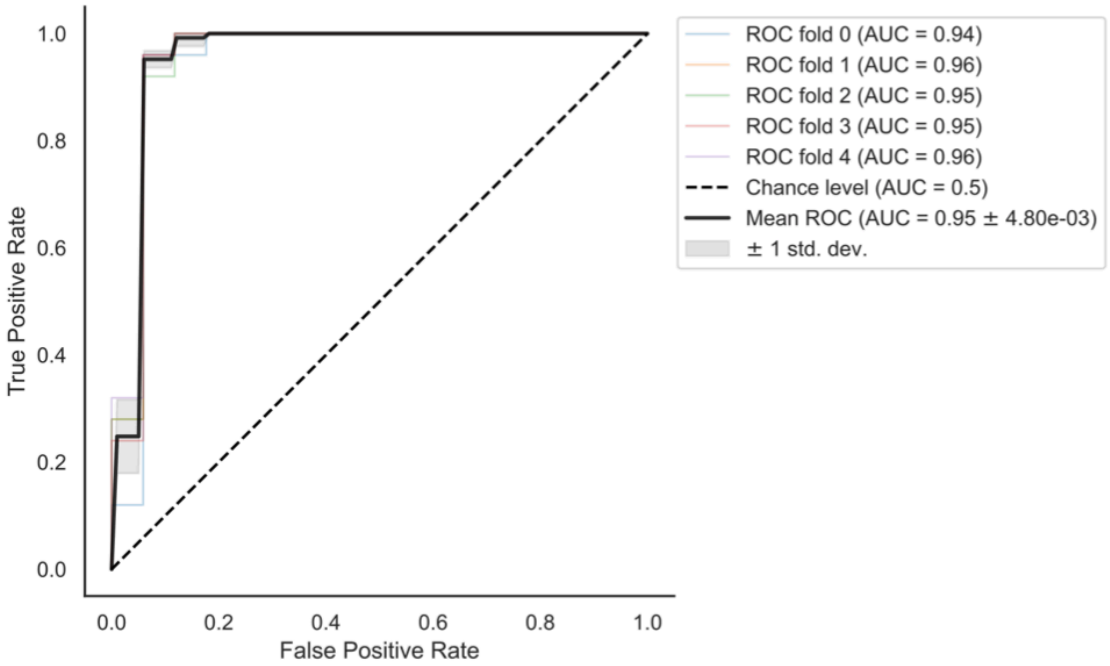

Receiver operating characteristics of the ACICU score (mean AUROC=0.95) in 5-fold cross- validation in test cohort.

Suppl. Figure S2: ACICU score in patients with ACLF without OLT

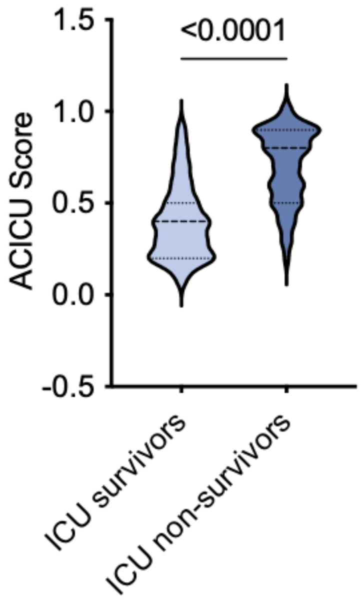

Comparison of ACICU score values between ICU survivors vs. ICU non-survivors after exclusion of patients who received OLT (68 vs. 119 patients), *p* < 0.0001. An unpaired, two-tailed t-test was used.

Suppl. Figure S3: ACICU score in patients with ACLF receiving OLT

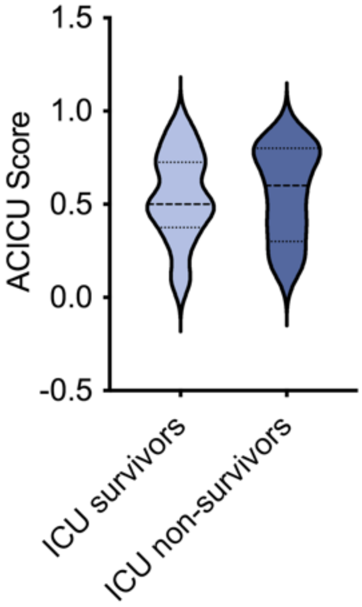

Comparison of ACICU score values between ICU survivors vs. ICU non-survivors after OLT out of ACLF (10 vs. 5 patients), p = 0.8849. An unpaired, two-tailed t-test was used.
